# The Enzymatic Decolorization of Textile Dyes by the Immobilized Polyphenol Oxidase from Quince Leaves

**DOI:** 10.1155/2014/685975

**Published:** 2014-01-21

**Authors:** Gulnur Arabaci, Ayse Usluoglu

**Affiliations:** Department of Chemistry, Faculty of Science and Arts, Sakarya University, Serdivan, 54187 Sakarya, Turkey

## Abstract

Water pollution due to release of industrial wastewater has already become a serious problem in almost every industry using dyes to color its products. In this work, polyphenol oxidase enzyme from quince (*Cydonia Oblonga*) leaves immobilized on calcium alginate beads was used for the successful and effective decolorization of textile industrial effluent. Polyphenol oxidase (PPO) enzyme was extracted from quince (*Cydonia Oblonga*) leaves and immobilized on calcium alginate beads. The kinetic properties of free and immobilized PPO were determined. Quince leaf PPO enzyme stability was increased after immobilization. The immobilized and free enzymes were employed for the decolorization of textile dyes. The dye solutions were prepared in the concentration of 100 mg/L in distilled water and incubated with free and immobilized quince (*Cydonia Oblonga*) leaf PPO for one hour. The percent decolorization was calculated by taking untreated dye solution. Immobilized PPO was significantly more effective in decolorizing the dyes as compared to free enzyme. Our results showed that the immobilized quince leaf PPO enzyme could be efficiently used for the removal of synthetic dyes from industrial effluents.

## 1. Introduction

Synthetic dyes are extensively used in many fields of industries, for example, the textile, leather, paper, rubber, plastics, cosmetics, pharmaceutical, and food [1]. The effluent of wastewater from these industries especially textile contains a variety of large quantities of dyes which are inert and may be toxic at the concentration discharged into receiving water. Many of these dyes which have complex aromatic molecular structures are also toxic and even carcinogenic and pose a serious threat to living organisms [2]. Toxic effects of industrial dyes encouraged researchers to continue studies on chemical and enzymatic methods to remove such hazardous materials [3, 4]. Compared to physicochemical methods such as precipitation, filtration, and absorption, the enzymatic treatments of dyes have low energy cost and are more ecofriendly, process not still commonly used in the textile industries [5].

Unfortunately, conventional wastewater treatments are ineffectual at removing dyes and involve high cost, formation of hazardous by-products, and intensive energy requirements. Moreover, complete dye removal is unfeasible. This has impelled research into alternative methods like biotechnological processes. Recently, enzymatic approach has attracted much interest in the removal of phenolic pollutants from aqueous solutions [6]. Oxidoreductive enzymes, polyphenol oxidases, and peroxidases are participating in the degradation/removal of aromatic pollutants from various contaminated sites [7, 8]. Polyphenol oxidases can act on a broad range of substrates such as substituted polyphenols, aromatic amines, benzenethiols, and a series of other easily oxidizable compounds. Thus, they can catalyze the decolorization and decontamination of organic pollutants. In view of the potential of the enzymes in treating the phenolic compounds, several microbial and plant oxidoreductases have been employed for the treatment of dyes, but none of them has been exploited at large scale due to low enzymatic activity in biological materials and high cost of enzyme purification. In order to improve polyphenol oxidases activity and stability, enzyme immobilization technology has been applied. This technology is an effective means to make enzymes reusable and to improve its stability, which is considered as a promising method for the effective decolorization of dye effluents. According to the previous reports, various types of supporters were applied to immobilize enzyme, such as activated carbon, celite, controlled porosity glass, chitosan microspheres, and alginate [9–13].

In this study, our first objective was to find a cheaper and easily available alternative plant polyphenol oxidase (PPO) enzyme source for the commercially available ones and its immobilization. Quince leaves (*Cydonia Oblonga*) which are waste in Turkey have been employed in this work as an easily available and inexpensive PPO enzyme source. PPO enzyme was partially purified from quince (*Cydonia Oblonga*) leaves and immobilized on calcium alginate beads. The biochemical properties were determined for free and immobilized quince (*Cydonia Oblonga*) leaf PPO. The second objective was to evaluate the performance of free and immobilized polyphenol oxidases regarding the decolorization of various reactive, acid, direct and basic dyestuffs.

## 2. Materials and Methods

### 2.1. Materials

Quince (*Cydonia Oblonga*) leaves, used in this study, were obtained from Sakarya region, Turkey, and stored at –20°C until used. Polyvinylpolypyrrolidone (PVP), (NH_4_)_2_SO_4_, Sodium alginate, CaCl_2_, and other chemicals were obtained from Sigma Chemical Co. and dyes were provided by kindly DyStar, Huntsman, and Yorkshire.

### 2.2. Extraction and Purification

30 g of quince (*Cydonia Oblonga*) leaves was obtained from local Sakarya region. The leaf samples were added to 50 mM sodium phosphate buffer (pH, 7.0), 0.5% g polyvinylpolypyrrolidone (PVPP), and 10 mM ascorbic acid and the mixture was homogenized with blender. After the filtrate was centrifuged at 14.000 g for 30 min, the supernatant was collected. Extraction was fractionated with (NH_4_)_2_SO_4_; solid (NH_4_)_2_SO_4_ was added to the supernatant to obtain 80% saturation. The mixture was centrifuged at 14,000 g for 30 minutes and the precipitate was dissolved in a small amount of phosphate buffer and then dialyzed at 4°C in the same buffer for 24 h with three changes of the buffer during dialysis. The dialyzed enzyme extract was collected and used for all other processes.

### 2.3. Enzyme Immobilization

Alginate solution (1, 2, 3%, w/v) was prepared by dissolving sodium alginate in deionized water. Crude quince leaf PPO solution was mixed with 20 mL of alginate solution at the enzyme/alginate ratio of 1 : 10 (v/v). The mixture was stirred with magnetic stirring to ensure that complete Ca-alginate beads were produced as soon as the emulsion was added into 100 mL 3 M CaCl_2_ (1, 2, 3%, w/v). The beads were allowed to harden for at least an hour under mild agitation. Then the Ca-alginate beads were removed from the encapsulation medium via centrifugation and rinsed twice with 0.5% (w/v) CaCl_2_ containing 1% (v/v).

### 2.4. PPO Activity Assay

The activity of free and immobilized PPO was determined at room temperature using catechol as a substrate. The assay mixture consisted of 2.95 mL of 20 mM catechol in 0.05 M potassium phosphate buffer pH 7.0 and 0.05 mL of enzyme. The increase in absorbance at 420 nm was measured as a function at time for 1 min. One unit of enzyme activity is defined as the amount of the enzyme that causes an increase in absorbance of 0.001 per min at 25°C. PPO activity was assayed in triplicate measurements.

For determining Michaelis constant (*K*_*m*_) and maximum velocity (*V*_max⁡_) values of the enzyme, PPO activities were measured with catechol at varying concentrations under optimum conditions of pH and temperature. *K*_*m*_ and *V*_max⁡_ values of PPO, for catechol substrate, were calculated from a plot of 1/*V* against 1/[*S*] by the method of Lineweaver and Burk [14].

### 2.5. Influence of pH

The activity assays were carried out over the pH range 3.5–9.0. Reaction rates of free and immobilized enzyme preparations depending on pH were investigated using 50 mM acetate buffer at pH 3.5, 4.0, 5.0, and 50 mM and phosphate buffer at pH 6.0, 7.0, 7.5, 8.0, and 9.0. Activity of pH profiles was determined at various pH values in 10 mM catechol solution at 25°C.

### 2.6. Influence of Temperature

The effect of temperature on enzyme activity was investigated in the range of 4–70°C for both free and immobilized PPO enzymes. Activity of temperature profiles was determined at indicated temperatures in 10 mM catechol solution (pH 7.0).

### 2.7. Effect of pH and Time on the Decolorization of Textile Dyes

Neutrilan Black MRX (C.I. Acid Black 194), Telon Turquoise M-5G (C.I. Direct Blue 86), Lanaset Yellow 4GN (C.I. Reactive Yellow 39), Telon Yellow ARB (C.I. Acid Orange 67), Isolan Grey NHFS (C.I. Acid Black 220), Telon Red MGWN, Solophenyl Red 7BE, and Astrazon Yellow 7GLL (C.I. Basic Yellow 21) dyes were selected for this study. Each dye was incubated with soluble and immobilized quince leaf PPO, 1.5 EU/mL in the buffers of varying pH values 4.0–7.0 at 25°C. Each dye was also incubated with immobilized quince leaf PPO, 1.5 EU/mL in pH 4.0 at 25°C for 30–60–90 min. Dye decolorization by PPO was monitored at the specific wavelength. The decolorization percentage was calculated by taking untreated dye solution as control of each buffer (100%).

### 2.8. Calculation of Dye Decolorization Rate

Starting absorbance at characteristic max for each dye (control) was designated as 100%. The extent of decolorization rate was defined by the following formula:

(1) $$\begin{array}{c} \text{Decolorization}\;\text{percentage}\;\left(\%\right)=\frac{\left(A_{o}-A\right)}{A_{o}}\times 100, \end{array}$$

where *A*_*o*_ is the absorbance of the untreated dye and *A* is the absorbance after treatment [15].

## 3. Result and Discussion

### 3.1. Effect of pH on Free and Immobilized PPO

**Figure 1:**
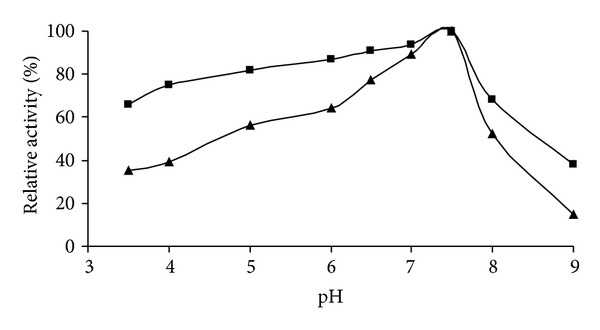
Effect of pH on activity: (▲) free PPO and (■) immobilized PPO.

For the determination of the effect of pH on free and immobilized enzymes by acid, phosphate buffers were used within the pH range of 3.5–9.0. The optimum pH for free and immobilized quince leaf PPO was 7.5 respectively (Figure 1). Both free and immobilized quince leaf PPO gave similar pH values. However, the immobilized leaf PPO gave much broader pH stability than the free enzyme. This suggests that immobilized quince leaf PPO was less sensitive to pH changes than the free one.

### 3.2. Effect of Temperature on Free and Immobilized PPO

**Figure 2:**
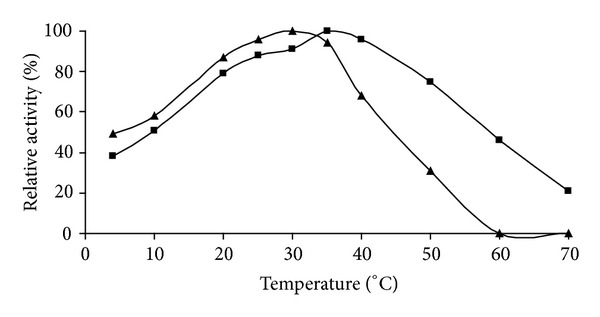
Effect of temperature on activity: (▲) free PPO and (■) immobilized PPO.

At the determination of the effect of temperature on free and immobilized quince leaf PPO activity was investigated in phosphate buffer in a temperature range of 4–70°C. The results showed that the optimum temperatures of the free and immobilized enzymes were 30°C and 35°C, respectively (Figure 2). The thermostability of immobilized quince leaf PPO enzyme was obviously better than free enzyme. The enhanced thermal stability of enzymes arising from immobilization would be an advantage for its industrial application due to the high temperatures used in the industrial processes [16].

### 3.3. Kinetic Properties

The Michaelis-Menten constants of free and immobilized quince leaf PPO were calculated by using Lineweaver-Burk double reciprocal models [14]. The calculated *K*_*m*_ values for free and immobilized quince leaf PPO were 5.86 and 12.57 mM respectively. An increase in the *K*_*m*_ value for catechol on the immobilization of PPO was observed. Our results are similar to the Michaelis-Menten constants of partially purified free and immobilized potato PPO. The values of *K*_*m*_ were 8.0 mmol L^−1^ for free potato PPO and 14.7 mmol L^−1^ for alginate SiO_2_/PPO, respectively. In general, *K*_*m*_ values of immobilized enzymes are higher than those for free enzymes, revealing an affinity change for the substrate [17].

### 3.4. Effect of pH on the Decolorization of Textile Dyes

The effects of pH on the decolorization of textile dyes by free and immobilized quince leaf PPO are summarized in Table 1. The effect of pH was studied at pH values between 4.0 and 7.0. The results showed that the decolorization rate was significantly higher at lower pH having maximum at pH 4.0 (Table 1). Also the immobilized PPO increased the decolorization percentage compared to the free enzyme. There were several earlier reports regarding the maximum decolorization of dyes by various plant polyphenol oxidases [17], plant peroxidases [18], microbial polyphenol oxidases [19], and laccases at acidic pH values [13–16]. When pH is increased above 7.0, the extent of decolorization was decreased rapidly. This is an advantage from industrial application point of view since some dye effluents are slightly acidic [19]. The results also showed that Telon Yellow ARB was the most effectively decolorized by immobilized quince leaf PPO at pH 4.0 with 72.68% decolorization.

### 3.5. Effect of Time on the Decolorization of Textile Dyes

**Figure 3:**
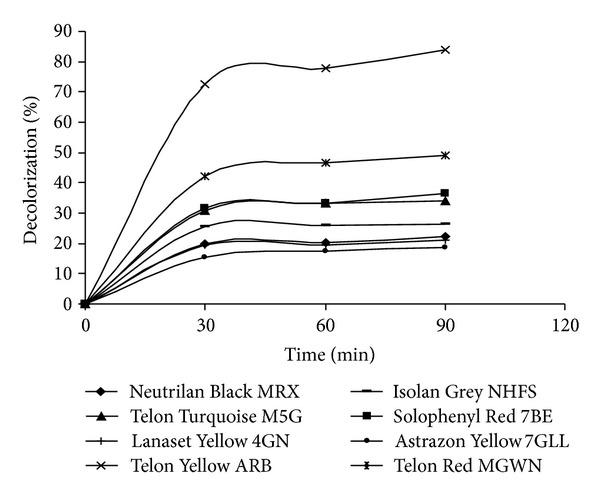
Removal of eight dyes by immobilized quince leaf PPO.

The enzymatic decolorization of textile dyes by immobilized quince leaf PPO was examined by varying the time of incubation (Figure 3). The eight different synthetic textile dyes were used for decolorization in this study. The results showed that the decolorization of dyes was increased with time up to 30 min. However, the rate of dye decolorization was quite slow after 1 h which may be probably due to products inhibition. This observation suggested that initial first hour was significant for dyes decolorization. These results were in agreement with earlier published work of decolorization of textile dyes [20, 21]. The most effected decolorization by immobilized quince leaf PPO was observed for the Telon Yellow ARB dye.

## 4. Conclusion

Our results showed that PPO enzyme was successfully partially purified from quince leaves and immobilized onto alginate beads. Immobilization of quince leaf PPO increased its stability to pH and temperature that could be more useful in industrial applications. Free and immobilized quince leaves PPO were applied to eight different textile dyes for decolorization. Immobilized quince leaf PPO enzyme was significantly more effective in decolorizing of the dyes as compared to free enzyme at pH 4.0. Our results clearly demonstrated that the different textile dyes in wastewater could be easily decolorized by the partially purified plant PPO obtained from cheaper plant sources like quince leaves. The use of partially purified immobilized quince leaf PPO may be extendable to the effluents coming out of industries and mixtures of dyes present in wastewaters.

## Figures and Tables

**Table 1:** Decolorization of textile dyes by quince leaf PPO.

Dyes	*λ*_max⁡_ (nm)	Immobilized PPO decolorization (%)		Free PPO decolorization (%)	
		pH: 4.0	pH: 7.0	pH: 4.0	pH: 7.0
Neutrilan Black MRX	570	19.69	16.39	12.23	10.85
Telon Turquoise M5G	616	30.7	20.53	8.65	3.3
Lanaset Yellow 4GN	398	19.62	13.1	5.25	2.75
Telon Yellow ARB	364	72.68	51.52	15.25	9.85
Isolan Grey NHFS	576	25.6	21.2	6.22	3.47
Solophenyl Red 7BE	515	31.53	28.76	21.27	18.89
Astrazon Yellow 7GLL	406	15.25	15.1	6.63	5.87
Telon Red MGWN	515	42.1	36.75	17.35	11.28
